# Demaghi, a polyherbal formulation, mitigates aluminum chloride-induced neurological impairment in mice: Insights from phytochemical analysis and behavioral assessment

**DOI:** 10.1016/j.heliyon.2023.e21234

**Published:** 2023-10-27

**Authors:** Hassan Ali, Hafiz Usman, Waseem Ashraf, Faleh Alqahtani, Sana Javaid, Farhan Siddique, Muhammad Fawad Rasool, Imran Imran, Tanveer Ahmad, Anas M. Abdel Rahman, Reem H. AlMalki

**Affiliations:** a Department of Pharmacology, Faculty of Pharmacy, Bahauddin Zakariya University, Multan, 60800, Pakistan; b Department of Pharmacology and Toxicology, College of Pharmacy, King Saud University, Riyadh, 11451, Saudi Arabia; c Department of Pharmacy, The Women University, Multan, 60000, Pakistan; d Departmenmt of Pharmaceutical Chemistry, Faculty of Pharmacy, Bahauddin Zakariya University, Multan, 60800, Pakistan; e Department of Pharmacy Practice, Faculty of Pharmacy, Bahauddin Zakariya University, Multan, 60800, Pakistan; f Institut pour l’Avancée des Biosciences, Centre de Recherche UGA / INSERM U1209 / CNRS 5309, Université Grenoble Alpes, France; g Metabolomics Section, Department of Clinical Genomics, Center for Genomics Medicine, King Faisal Specialist Hospital and Research Centre (KFSHRC), Riyadh, 11211, Saudi Arabia; h Department of Botany and Microbiology, College of Science, King Saud University, Riyadh, 11451, Saudi Arabia

## Abstract

Herbal products have been very popular in Pakistan for their curative significance against various disorders. Demaghi (DEMG) is a widely used herbal product claimed to own natural substances having neuroprotective potential. The current study aims to scientifically validate the chemical composition as well as its neuroprotective claims of this widely used herbal tonic. The commercially available Demaghi product was chemically characterized for its phytocomposition. The mice were treated with two doses of Demaghi (DEMG 50 mg and 100 mg/kg/day), and the effects of its prolonged exposure on animal anxiety, memory, and depression were noted through a series of behavioral tests in the AlCl_3_-induced memory deficient mice model. Besides that, dissected brains were biochemically analyzed for oxidative stress markers and acetylcholinesterase activity, as well as histopathological changes. The study outcomes showed that DEMG (100 mg/kg/day) has prominent anti-anxiety effects, memory-enhancing properties, and anti-depressants effects observed in the AlCl_3_-induced memory-deficient mice model. Biochemical assays also showed a greater decrease in oxidative stress of tested animals treated with 100 mg/kg/day of DEMG. The histopathological analysis also revealed that administration of DEMG reduced the AlCl_3_-induced toxicity. UPLC-MS results revealed the presence of many phytoconstituents, which showed to support cholinergic signaling in *in-silico* studies. The current research validates the neurological benefits of Demaghi for memory-boosting properties. The phytocompounds present in Demaghi exert neuroprotective effects, possibly by enhancing the cholinergic neurotransmission and combating the neurotoxin-induced oxidative stress.

## 1 Introduction

The neurological ailments affect approximately one in every six individuals irrespective of one's age, gender, income and education. Alzheimer's disease (AD) is one of the major neurological ailments typically affecting people above 65 years of age and is thought to be responsible for 50–60 % of dementia cases [1]. According to research, the incidence increases dramatically with age, rising from 3 % in patients between the ages of 65 and 74 years to as high as 47.2 % in patients over 85 years [2]. This condition is characterized by progressive memory loss, a decline in nearly all intellectual abilities, increased apathy, and impaired speech function [3]. These neurodegenerative changes during progression of this disease cause cognitive insufficiency eventually affecting the quality of individual's life resulting in his life-long physical dependency on others [4].

Aluminum chloride (AlCl_3_) is a neurotoxic substance that is commonly present in the environment and its toxicity precipitates various neurodegenerative illnesses like AD, anxiety, and depression [5]. AlCl_3_ can cross the blood-brain barrier by help of specific high-affinity receptors, causing cognitive impairment by decreasing cholinergic function and producing oxidative stress [6]. One crucial mechanism behind development of AlCl_3-_induced AD is increased oxidative stress, which leads to neuronal apoptosis and neurodegeneration [7]. Oxidative stress alters cellular function, suppresses the antioxidant defense system and causes structural damage to functional proteins in the brain. Additionally, AlCl_3_ causes inflammatory reactions and enhances cellular proteins such as neurofilaments, microtubule-associated proteins, and phosphorylated cytoskeletal proteins leading to Alzheimer's disease [8].

Cholinesterase inhibitors are widely used as therapeutic options to manage AD, which tackle the issue of declining memory in patients by improving the cholinergic neurotransmission [9]. Unfortunately, these agents' use has limitations due to their high prices, poor safety profiles, and shorter half-lives. Thus, in an attempt to look for alternative remedial options, people in Pakistan have developed an interest in using various herbal products. These commercially available natural preparations are gaining popularity as these price-friendly products are claimed to possess a variety of natural substances with little or no side effects [10].

The inadequate availabilty and poor affordability of therapeutic remedies cause majority of population to depend on phtycomponents comprising herbal remedies for different disorders in developing countries. Demaghi, an herbal product made by Qarshi Industries, claims to improve cognitive and other physiological brain functions and is suggested to be taken with breakfast or dinner. It is in the form of semi-solid edible paste having 22 components derived from plant and mineral sources, and every 10 g of Demaghi contains Coral compounds (131.87 mg), Borage (82.41 mg), Clary sage (59.34 mg), Leopards bane (59.34 mg), White behen (59.34 mg), Indian catmint (59.34 mg), Coriander (59.34 mg), Rose (32.97 mg), Cinnamon (32.97 mg), White Sandalwood (32.97 mg), Lichen (32.97 mg), Emblic (32.97 mg), Purslane (32.97 mg), Borage flowers (32.97 mg), Bamboo manna (32.97 mg), Barberry fruit (32.97 mg), Olibanum gum (32.97 mg), Cooling seeds (26.37 mg), Virginia Peppergrass (32.97 mg), greater Cardamom seed (16.48 mg), Screw pine (10 mg). The weights mentioned are of dried herbs and minerals before extraction, processing and blending in palatable base. Detailed information about the ingredients is provided in the supplementary data (Composition of Demaghi). Many of these components have been reported previously to posess neuroprotective capabilities, and a cocktail of these components can either increase their therapeutic value or alter their personal effects.

*Coriandrum sativum* is well known to have anxiolytic and memory-improving characteristics [11]. Emblic is an effective herbal medication used to treat a variety of neurological problems [12] as *Emblica officinalis* use reversed scopolamine-induced amnesia in a dose-dependent manner resulting in improved memory and cognition [13]. Cinnamon is a popular spice used daily around the world [14] and has been reported to exert beneficial effects against neurological ailments including Alzheimer's and Parkinson's disease through its anti-inflammatory and antioxidant potential [15]. Furthermore, purslane, used as a nutritional supplement, is rich in minerals and omega-3 fatty acids with memory-enhancing properties as its use in experimental rats protected from d-galactose-induced toxicity and behavioral improvement [16].

The present study has been designed to scientifically validate the neuroprotective claims of Demaghi, which is widely used and locally manufactured by Qarshi Industries (Pvt) Ltd., Pakistan. In the current study, this commercially available herbal tonic was chemically characterized for its phytocomposition followed by chronic administration to mice subjected to aluminum chloride-induced AD. The treated mice were tested for neurobehavioral parameters of anxiety, cognition, and depression with the subsequent biochemical and histopathological assessment of isolated brains.

## 2 Materials and methods

### 2.1 Drugs and chemicals

Demaghi from Qarshi Industries (Pakistan), donepezil from Acros Organics, and aluminum chloride from Merck were procured. After dissolving in water, aluminum chloride (200 mg/kg/day) was given *ad libitum* to test mice via water bottles placed in their cages for 28 days [17]. Donepezil was dissolved in distilled water and injected by the intraperitoneal route (i.p.) at the dose of 5 mg/kg/day [18]. Demaghi 50 and 100 mg/kg/day were mixed into a normal diet and fed to the test mice for 28 days.

### 2.2 Animals and their housing

Male BALB/c mice (10–12 weeks old) weighing 30–35 g were used in this study. The animals were kept in a sanitary environment at 25 °C in animal house at the Faculty of Pharmacy, Bahauddin Zakariya University, Multan. A 12 h light/dark cycle was maintained and all animals were fed with a diet rich in protein and carbohydrates. All experiments were conducted in accordance with the permission granted by Department of Pharmacology Ethical Committee at 10.13039/100007713Bahauddin Zakariya University Multan (04-PHL-S21).

### 2.3 Grouping and dosing of animals

This study comprised seven groups (n = 8), and eight animals were kept per cage. The animal groups included the **healthy control** (which received regular food and water), **AlCl**_**3**_**-control** (which received aluminium chloride 200 mg/kg/day orally), **Donepezil** + **AlCl**_**3**_ (received aluminium chloride and the conventional drug donepezil 5 mg/kg/day, i.p. simultaneously), **DEMG 50** (received food pellets containing the herbal drug Demaghi 50 mg/kg/day), **DEMG 100** (received food pellets containing the herbal drug Demaghi 100 mg/kg/day), **DEMG 50** + **AlCl**_**3**_ (received Demaghi 50 mg/kg/day along with AlCl_3_ 200 mg/kg), **DEMG 100** + **AlCl**_**3**_ (received Demaghi 100 mg/kg/day along with AlCl_3_ 200 mg/kg).

**Fig. 1:**
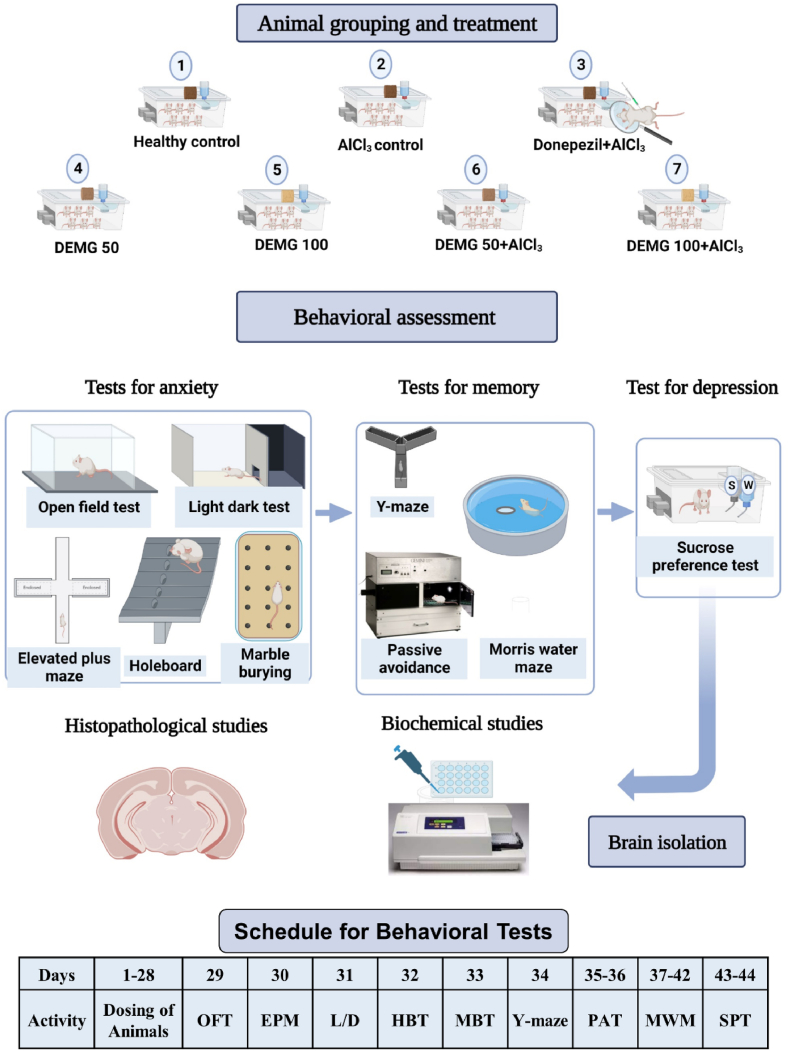
Experimental design for current study. The animals were divided into seven groups and provided with group-designated treatments for 28 days. After this, the impact of DEMG was tested on animal's behavioral changes by allowing animals to perform in a series of experimentation to evaluate their anxiety, cognition and depression-like behavior. Immediately after behavioral tests, animals were decapitated for brain isolation to evaluate the isolated brains in biochemical and histopathological studies The illustration of experimental scheme has been created through Biorender.com (LO25HJL15E; Dated June 14, 2023). OFT: Open Field Test, EPM: Elevated Plus Maze, L/D: Light and Dark Test, HBT: Hole Board Test, MBT: Marble Burying Test, PAT: Passive avoidance test, MWM: Morris water maze test, SPT: Sucrose preference test.

All the experimental groups received treatment for 28 days except the healthy control group. From day 29, animals were tested in a series of behavioral experiments from 8:00 a.m. to 6:00 p.m. Animals were acclimatized to the environment of the behavioral room and the experimenter's handling before each behavioral experiment. All behavioral tests were video-recorded by using Logitech camera and analyzed by ANYmaze software (trial version 7.1). The complete experimental layout for current study has been presented in Fig. 1.

### 2.4 Tests for anxiety

#### 2.4.1 Open field test (OFT)

This test is designed to evaluate rodents' anxiety and locomotor activity. It comprises square-shaped equipment (45 cm × 45 cm) with high plastic walls that are made of polyacrylic sheets. The floor of the equipment is separated into two zones by smaller squares (10 × 10 cm). Immediately following the completion of the treatment for 28 days, each mouse was positioned in the open field on 29th day of study and observed for its exploratory activity for 300 s. The animal was then removed and the equipment was sprayed with a 70 % isopropyl alcohol solution before next animal's trial [19]. The any-maze software was used to examine and evaluate the experimental videos. In order to measure the animal's anxiety, animals were noted for the variables including number of central zone visits and amount of time spent in that region. To evaluate the impact of demaghi on animal's locomotion, the animal's total distance traveled and line crossings were monitored.

#### 2.4.2 Elevated plus maze (EPM)

This experiment evaluates the anxiety-related behavior of mice as rodents innately avoid the open regions and spend more time in hidden areas. The equipment utilized for this test is made up of uplifted arms that form a plus-shaped (+) symbol. The central platform was elevated 46 cm above the ground by two open arms (14.5 × 5.5 cm) and two closed arms (14.5 × 5.5 cm). On 30th day, individual mouse was placed in the middle of the plus-shaped apparatus and was given 300 s to explore the all four arms of apparatus. The anxiety was evaluated by noticing the entries in open arms and time spent there [20].

#### 2.4.3 Light and dark (L/D) test

Through L/D test, rodents are examined for their anxious behavior as they naturally desire to discover new environments and avoid highly illuminated open regions [21]. On 31st day of study, the L/D experiment was carried out in a two-dimensional apparatus (21 × 21 × 25 cm) that had two connected compartments, one of which was highly lighted and the other of which was completely dark, and these two boxes share an opening route, which makes it simpler for mice to migrate from one to another. To assess the time that animals spent in light and dark areas, each mouse was introduced in illuminated box and allowed to freely explore both boxes of apparatus for 300 s. The preference for illuminated zone was considered as an indication of reduced anxiety-like behavior.

#### 2.4.4 Hole board test (HBT)

The experimental apparatus had small circular holes at the bottom of the experimental area. A head-dipping in the holes was an important hallmark of this behavioral test. On 32nd day, every mouse was individually introduced in the middle of the apparatus (40 cm × 40 cm) comprising 16 equidistant holes of 2.5 cm diameter and permitted to investigate the area for 5 min. Because mice have an intrinsic desire to discover new areas, the tested mice drop their heads into the holes. The frequency of head-dipping in the holes was monitored and the increased number of head dipping was taken as a sign of reduced anxiety [22].

#### 2.4.5 Marble burying test (MBT)

This test is designed to assess the natural aptitude of rodents to dig and bury tiny things. Animals' marble-burying behavior is thought to be related to their defensive traits and nervousness. On 33rd day, fifteen marbles were dispersed uniformly across two-thirds of the cage space comprising 5 cm thick layer of sawdust. The animals were then individually placed into the cages and permitted to engage with the marbles for 30 min. The mice were then carefully retrieved from their cages when the testing was finished, and a photo was captured to evaluate the marble burying. Marble was considered buried if the sawdust bedding material covered 90 % of its surface [23]. In this manner, the number of marbles 2/3 covered with sawdust was counted for statistical analysis. The test animals who felt relaxed and did not exhibit any signs of anxiety left the marbles untouched, whereas the agitated test animals buried more marbles.

### 2.5 Tests for learning and memory

#### 2.5.1 Y-maze test

The spatial working memory of rodents is examined using an apparatus comprising three equal arms (40 × 7.5 × 14.5 cm) to make a Y-shaped maze. On 34th day of study, all mice were individually positioned in one arm of the apparatus and given 5 min to investigate all three arms (A, B and C) of the maze. Because of the inbuilt characteristics of rodents to prefer the new environment, they tend to enter the arm which was not recently explored resulting in alteration i.e., ABC, ACB, BCA, etc [24]. The following formula was used to compute the percentage of spontaneous alteration:

Percentage of spontaneous alteration = (Number of alterations/total no. of arms entries – 2) × 100.

#### 2.5.2 Passive avoidance test (PAT)

In this test, animals were tested for their memory of aversive stimuli given in an innately preferred apparatus compartment. The apparatus has two chambers (9 × 7.5 × 7.5 cm), one of which is light and the other of which is dark. An automated gate links these two sections together. A 50 W light source is used to brightly illuminate the light compartment, while a stainless-steel grid floor is used in the dark section. The animals receive an electric shock of 0.5 mA for 2 s as an unpleasant stimulus. Mice were introduced individually into the illuminated compartment throughout the training period. Due to innate preference for dark areas, mice frequently move toward the dark chamber as the gate opens. On 35th day, the animals were given an electric shock when they entered the dark chamber to help them remember this undesirable experience. After 1 h (the short-term memory formation capability tested on 35th day) and 24 h (the long-term memory retention capacity tested on 36th day), all of the animals got the same treatment for 300 s without receiving an electrical shock, and step-through latencies were recorded for each test animal to assess memory and learning capacity [25].

#### 2.5.3 Morris water maze (MWM) test

This test was incorporated to assess the cognitive functions of rodents by evaluating their spatial learning capability. The MWM apparatus comprises of a large, round water tank with dimensions of 100 cm wide and 60 cm in height. The tank is often filled with ordinary water and kept at room temperature. There were four divisions in the water tank: north, east, south, and west. NE, NW, ES and SW were the names of these quadrants, and one of them included a square platform (12 × 12 cm) so that the water level was 2 cm lower than the platform and 2 cm higher on test days. To help the tested mice locate the platform, four hints or points of different shapes were placed outside the tank. Within the walls of a round tank, proximal indicators of the same color and shape were positioned. Throughout the MWM test, these indicators persisted in their original locations.

The MWM procedure is a 6-day test which was carried out from 37th-42nd days of study. The six days of MWM test consisted 2 days of training, 3 days of experiments, and one day for probe trial [19]. During training days, three trials were done and each trial was completed in 120 s assuming the tested mice could easily reach the destination platform, positioned in the south-west quadrant of the maze. As during that phase, each training should be delivered from a separate quadrant. One trial from each zone was conducted daily during the experimental trials, and excessive noise was reduced to ensure that it did not affect animal behavior. On the probe day, platform was removed and mice were allowed to swim in entire water maze for 2 min. To evaluate spatial memory, the number of animal's entries and swimming duration in the specified platform area were calculated.

### 2.6 Tests for depression

#### 2.6.1 Sucrose preference test (SPT)

SPT is a reward-based test that analyzes depression-like behavior in rodents. On 43rd day, each test mouse was provided free access to two bottles after a 12-h fast. One of the bottle contained ordinary water (120 mL) while the other bottle contained 1 % sucrose solution in water (120 mL). The quantity of 1 % sucrose solution consumed over 24 h was noted on 44th day of study [26]. When rodents prefer regular water over sucrose solution, it indicates that they are fearful and anxious.

The percentage of sucrose preference (%SP) swas calculated as follows:

%SP=(volumeof1%sucroseconsumed)÷(volumeof1%sucroseconsumed+volumeofwaterconsumed)×100

### 2.7 Biochemical analysis

#### 2.7.1 Preparation of tissue homogenate

From all groups, animals (n = 4) were immediately decapitated to dissect their brains. After rinsing all isolated brains with 7.4 pH phosphate buffer saline (Solarbia, Life Sciences), weighed brain was mixed with its 10 times volume of PBS (1:10 w/v) to thoroughly homogenize the brain tissue [27]. The mixture was then placed in microcentrifuge tubes and centrifuged for 10 min at 12000 rpm to obtain clear supernatant. Aliquots of the homogenate were then stored at −40 °C for enzymatic tests while the pellet was removed.

#### 2.7.2 Malondialdehyde assay

MDA was determined by taking 200 μL of tissue homogenate. Then a solution of 100 μL of thiobarbituric acid (TBA) (Uni-chem Chemical Reagents) and 100 μL of trichloroacetic acid (TCA) (Uni-chem Chemical Reagents) in a 1:1 ratio was introduced in a microcentrifuge tube. The reaction mixture was heated in a microcentrifuge tube in a water bath for 15 min, then cooled to room temperature, and later centrifuged for 10 min at 3500 rpm [26]. The blank was similarly made, but without the homogenized brain. The absorbance of this mixture was measured in a microplate reader (Spectramax 340 PC384 by Molecular Devices, USA) at 532 nm, and the MDA concentration was estimated as nano-moles/mg of protein.

#### 2.7.3 Superoxide dismutase (SOD) assay

SOD was determined by adding brain tissue homogenate (50 μL), sodium carbonate solution (50 μL) (Sigma, Aldrich), nitro blue tetrazolium (NBT) (40 μL) (Molekula, England) and EDTA (20 μL) (Sigma, Aldrich) in a microcentifuge. The mixture was combined with 40 μL of hydroxylamine hydrochloride to begin the reaction that turns NBT into formazan [28]. The blank was similarly made, but without the brain tissue homogenate. To assess the kinetics of the enzymes, the samples were transferred into a microplate reader (Spectramax 340 PC384 by Molecular Devices, USA) and the absorbance at 570 nm was recorded at intervals of 0, 5, 10, 15, and 45 min.

#### 2.7.4 Catalase assay

Catalase activity was determined by adding 50 μL PBS, 40 μL H_2_O_2_, and 10 μL tissue homogenate to a microcentrifuge tube. Following that, the solution was kept for 90 min at 37 °C. 100 μL of potassium dichromate acetic acid (5 % solution) was added to terminate the process as the color turned blue [29]. The solution was then boiled for 15 min at 100 °C for formation of chromic acetate indicated by change in color from blue to green. Finally, the absorbance was measured at 570 nm after centrifuging at 2500 rpm for 5 min.

#### 2.7.5 Glutathione peroxidase (GPx) assay

The glutathione peroxidase activity was measured by combining 20 μL of homogenized brain, 10 μL of hydrogen peroxide (H_2_O_2_) (Sigma, Aldrich), 20 μL of PBS, 20 μL of reduced glutathione (Oakwood chemicals), and 10 μL of sodium azide in a microcentrifuge tube, and incubation at 37 °C for 15 min. 40 μL of 10 % trichloroacetic acid was added, and the mixture was then swirled at 1500 rpm for 5 min [27]. Then, 100 μL of this solution was combined with 70 μL of 5,5′-dithio-bis-2-nitrobenzoic acid (DTNB) and 20 μL of 0.8 mM ethylenediamine tetraacetic acid, and the absorbance of this mixture was calculated in a microplate reader (Spectramax 340 PC384 by Molecular Devices, USA) at 420 nm.

#### 2.7.6 Acetylcholinesterase (AchE) assay

To measure AchE activity, 40 μL of tissue homogenate, 20 μL of 0.01 M DTNB, and 138 μL of PSB were mixed in 1.5 mL microcentrifuge tube. The reaction mixture's basal reading was measured at 412 nm followed by addition of 2 μL of a solution of acetylcholinethioiodide resulting in yellow color [30]. Later absorbance at 412 nm was recorded every 2 min in a microplate reader from 0 to 10 min. The blank was similarly made, but without the brain tissue homogenate.

### 2.8 Histopathological examination

For histopathological examination, two animals were sacrificed from the represented groups and the brains were extracted after transcardial perfusion. The isolated brains were fixed by 4 % formaldehyde solution in PBS for 48 h at 4 °C and later embedded in paraffin for sectioning through microtome. Sections of hippocampus (of 5 μm thickness) were later stained with 0.1 % cresyl violet (Alfa Aesar, Thermo Fischer Scientific, USA) for 20 min and later dehydrated and covered with DPX new (Sigma-Aldrich) and coverslip.

### 2.9 Ultra performance liquid chromatography -mass spectrometry (UPLC-MS) analysis

The small molecules were extracted from the polyherbal using 40:40:20 Methanol: Acetonitrile: Water and the phytochemical constitutents profile was collected using the Waters ACQUITY ultra performance liquid chromatography system coupled to a Xevo G2-S QTOF mass spectrometer (Waters Corporation, USA) with an electrospray ionization source [31]. The solution was introduced to an ACQUITY UPLC using XSelect (100 × 2.1 mm, 2.5 μm) column (Waters Ltd., Elstree, UK). Mobile phase A contained 0.1 % formic acid in dH_2_O and mobile phase B 0.1 % formic acid in 50 % Acetonitrile: Methanol. The phytochemical constituents were chromatographed in a gradient elution as follows: 0–16 min 95- 5 % A, 16–19 min 5 % A, 19–20 min 5–95 % A, 20–22 min 95- 95 % A, at flow rate of 300 μL/min. Mass spectra were obtained under positive electrospray ionization (ESI+) mode. The eluted components were ionized at a temperature source of 150 °C; the desolvation temperature was 500 °C, a capillary voltage of 3.20 kV, cone voltage of 40 V, and desolvation gas flow was 800.0 L/h, and cone gas flow of 50 L/h. The collision energy of both low and high functions was placed at 0 and 10–50 V, respectively, in MSE mode. The mass spectrometer was calibrated with sodium formate in 100–1200 Da. Data were collected in continuum mode with Masslynx™ V4.1 (Waters Technologies, Milford, MA., USA) workstation.

#### 2.9.1 Phytochemical constituents identification

Peak selection and alignment of detected ions (*m*/*z*, Rt) were done by Progenesis QI v.3.0 software (Waters Technologies, Milford, MA., USA) [32]. Data were log-transformed, mean-centered, and Pareto-scaled for univariate analyses. Phytochemical constituents of the formulation were putatively annotated based on the exact mass, fragmentation pattern, and isotopic distribution search against the PlantCyc database https://plantcyc.org/, Planta Piloto de Química Fina. Universidad de Alcalá database, NIST database, NIST spectra, and NIST Chemistry WebBook.

### 2.10 Molecular docking methodology

#### 2.10.1 Selection of target protein

The acetylcholinesterase (AChE) protein was selected as the target protein based on its properties and activities. The protein structure with PDB ID 4EY7 was obtained from the RCSB website. The undesired chains were removed during the protein preparation wizard module's review phases, and only the chains containing donepezil were retained.

#### 2.10.2 Preprocessing of protein structure

The Maestro platform's Protein Preparation Wizard module was used to prepare the protein structure [[33], [34], [35]]. The bond orders were assigned, hydrogens were added, zero-order bonds were formed to the metals and di-sulphate bonds, seleno-methionines were changed into methionine, and the missing side chains were filled in. The structure was opitimized using energy minimization and hydrogen bond optimization by OPLS4 force field [36].

#### 2.10.3 Preprocessing of ligand structures

Three-dimensional structural data files (3D SDF) for the target ligands (1–12) were retrieved from PubChem databases. The LigPrep module was used to prepare the ligand structures. The structures were also energy minimized using the OPLS4 force field [36], producing 32 distinct states of stereoisomeric and tautomeric entities.

#### 2.10.4 Docking

Docking of the target proteins and ligand molecules was done by the Maestro platform's Glide docking module [[33], [34], [35]]. The Glide XP (extra precision) mode was used for docking. The XP pose viewer was used to analyze the docked ligand and protein interactions and obtain the optimal pose. The ligand interaction module generated a 2D interaction diagram of the ligand-protein complex molecule which was further analyzed to explore the interaction between the target protein and ligand molecules during the binding process through resulting XP posture.

#### 2.10.5 Validation

The docked complexes were validated using appropriate statistical methods, such as root-mean-square deviation (RMSD) calculations, Glide biding energy and Glide G score.

### 2.11 Statistical evaluation

Statistical evaluation of the data was done by GraphPad Prism 8. All behavioral and biochemical data were evaluated using one-way ANOVA followed by the Dunnett test, except for the escape latencies reported in the passive avoidance and water maze test, which were assessed with the help of two-way ANOVA followed by Tukey's test. All data was displayed as mean ± standard error mean. If P < 0.05, the outcomes were regarded as statistically significant.

## 3 Results

### 3.1 OFT

**Fig. 2:**
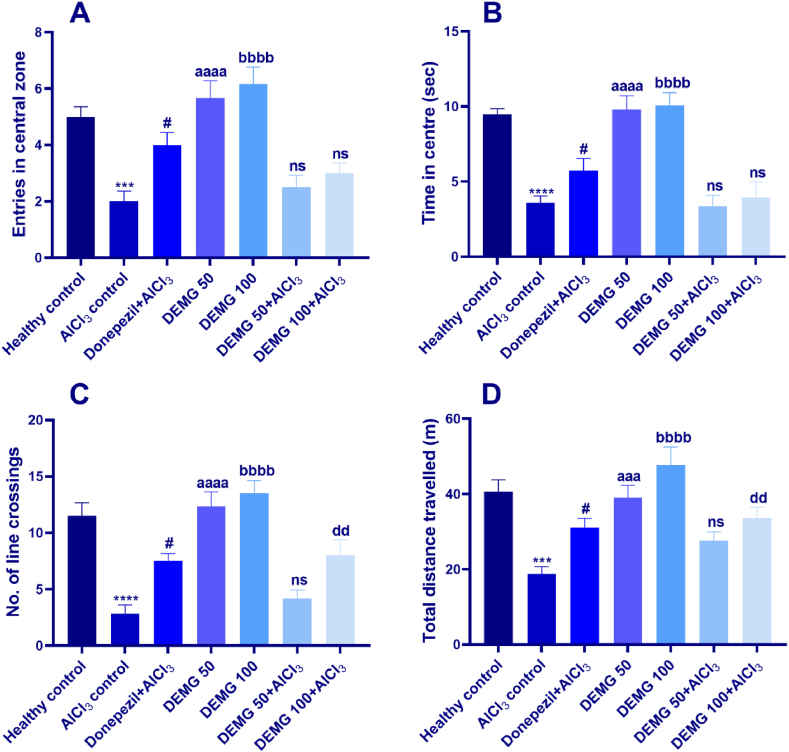
The impact of Demaghi on anxiety-related activity in an open field test. After treatments with DEMG alone and with simultaneous administration of AlCl_3_, the anxiety-like behavior and locomotor activity were assessed by monitoring the mice for 5 min in the open field to note their (A) entries in the central zone (B) time in the central zone (C) no. of line crossings, and (D) total distance traveled. All data were described in mean ± S.E.M (n = 6) while statistical analysis was performed by one-way ANOVA preceded by Dunnett Test comparing all groups with AlCl_3_ treated animals. ***P˂0.001, ****P˂0.0001 comparison between healthy control and AlCl_3_ treated group, ^#^P˂0.05 comparison between Donepezil + AlCl_3_ and AlCl_3_ group, ^aaa^P˂0.001, ^aaaa^P˂0.0001 comparison of DEMG 50 with AlCl_3_ control, ^bbbb^P˂0.0001 comparison between DEMG 100 and AlCl_3_ control, ^dd^P˂0.01 comparison of DEMG 100+AlCl_3_ with AlCl_3_ treated group while ns shows non-significant outcomes.

To evaluate locomotion and anxiety-like behavior, the mice were allowed to explore the square-shaped open arena. The animal's preference for the central region was monitored by their entries in the central area and the time spent in this region. The one-way ANOVA demonstrated that entries in the central region were statistically different among the groups [F (6,35) = 12.04, P < 0.0001] as well as time in the center also varied noticeably among all groups [F (6,35) = 16.05, P < 0.0001]. In detail, AlCl_3-_treated amnesic control showed significantly increased anxiety-like behavior (P < 0.001) compared to healthy control. Mice treated with DEMG at both doses (50 and 100 mg/kg/day) caused a significant reduction in anxiety towards the open arena as these animals entered the central area more frequently (P < 0.0001) and spent more time in exploration of the central zone of the maze (P < 0.0001), as compared to AlCl_3-_treated amnesic control. However, the DEMG at both doses could not produce significant anxiolytic benefits in animals co-administered with DEMG and AlCl_3_ as shown in Fig. 2A and B.

By determining the total distance traveled and the no. of line crossings, the OFT was also used to estimate the impact of the administration of DEMG on animal locomotion. In comparison to the healthy control, the disease-induced mice showed a reduced number of line crossing (P < 0.0001) and traveled shorter distances (P < 0.001). Animals receiving DEMG at doses of 50 and 100 mg resulted in increased line crossings (P < 0.05) (Fig. 2C) and distance traveled (P < 0.05) (Fig. 2D). Moreover, the DEMG at a dose of 100 mg significantly protected (P < 0.01) the mice from AlCl_3_-induced reduction in both parameters of locomotor activity, i.e., line crossings and distance traveled, revealing that prolonged administration of DEMG might exert beneficial effects on the brain.

### 3.2 EPM

**Fig. 3:**
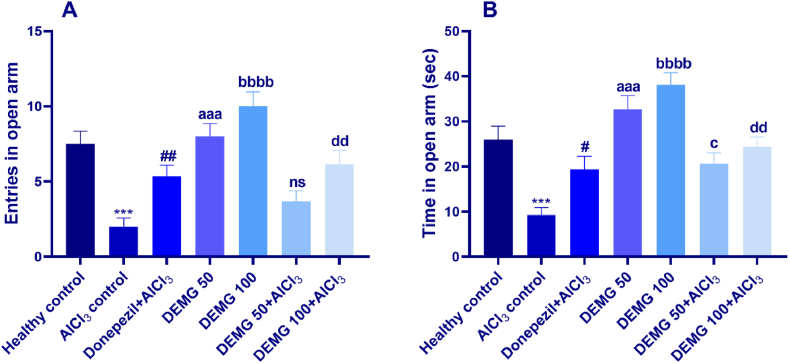
The impact of Demaghi on anxiety-related activity in an elevated plus maze test. After treatments with DEMG alone and with simultaneous administration of AlCl_3_, the anxiety-like behavior was evaluated by monitoring the mice for 5 min in maze to note their (A) entries in open arm and (B) time in open arm. All data were described in mean ± S.E.M (n = 6) while statistical analysis was performed by one-way ANOVA preceded by Dunnett Test comparing all groups with AlCl_3_ treated group. ***P˂0.001 comparison between healthy control and AlCl_3_ treated group, ^#^P˂0.05, ^##^P˂0.01 comparison among Donepezil + AlCl_3_ and AlCl_3_ control, ^aaa^P˂0.001 comparison of DEMG 50 with AlCl_3_ control, ^bbbb^P˂0.0001 comparison between DEMG 100 and AlCl_3_ control, ^c^P˂0.05 comparison between DEMG 50+AlCl_3_ and AlCl_3_ control^, dd^P˂0.01 comparison between DEMG 100+AlCl_3_ and AlCl_3_ control while ns shows non-significant outcomes.

The anxiety-like behavior in mice was further evaluated by permitting them to discover a plus-shaped maze lifted from the ground. The one-way ANOVA displayed a significant inter-group variation in entries in open arms [F (6,35) = 11.11, P < 0.0001] time spent in open arm [F (6,35) = 12.99, P < 0.0001]. The mice of the AlCl_3_-treated amnesic group showed significantly increased anxiety as their entries and duration of stay were notably reduced (P < 0.001) compared to healthy animals. The long-term treatment of DEMG alone at doses of 50 and 100 mg/kg/day showed dose-dependent anxiolytic effects as their entries and duration of open arm exploration were increased with P < 0.001 (DEMG 50) and P < 0.0001 (DEMG 100) when compared with AlCl_3_ group as shown in Fig. 3A and B. Furthermore, the co-administration of AlCl_3_ and DEMG showed significant protection from anxiety, as DEMG 100 caused an increase in entries (P = 0.0048). In contrast, duration in the open arms was increased in a dose-dependent manner with P = 0.0187 (DEMG 50+AlCl_3_) and P = 0.001 (DEMG 100+AlCl_3_), revealing that DEMG protected the rodents from the neurotoxic effects of aluminium chloride.

### 3.3 L/D box

**Fig. 4:**
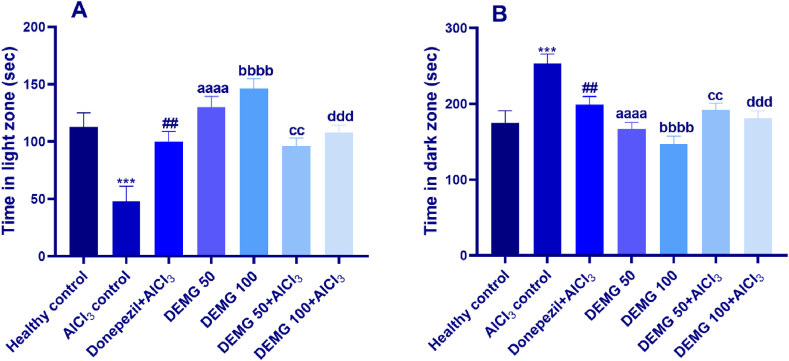
The impact of Demaghi on anxiety-related activity in light and dark box test. After treatments with DEMG alone and with simultaneous administration of AlCl_3_, the anxiety-like behavior was evaluated by monitoring the mice for 5 min in the test apparatus to note their (A) time in light area and (B) time in dark area. All data were described in mean ± S.E.M (n = 6) while statistical analysis was performed by one-way ANOVA preceded by Dunnett Test comparing all groups with AlCl_3_ control. ***P˂0.001 comparison among healthy control and AlCl_3_ control, ^##^P˂0.01 comparison between Donepezil + AlCl_3_ and AlCl_3_ control, ^aaaa^P˂0.0001 comparison between DEMG 50 and AlCl_3_ control, ^bbbb^P˂0.0001 comparison between DEMG 100 and AlCl_3_ group, ^cc^P˂0.01 comparison between DEMG 50+AlCl_3_ and AlCl_3_ control^, ddd^P˂0.001 comparison between DEMG 100+AlCl_3_ and AlCl_3_ control.

The results of the L/D box test revealed a significant variation between groups in terms of time spent in the light [F (6,35) = 8.88, P < 0.0001] and dark and [F (6,35) = 10.54, P < 0.0001] zones. The *post hoc* test demonstrated that in comparison to the healthy group, AlCl_3_-treated animals showed reduced courage to explore the illuminated areas of apparatus, anxiogenic for rodents, resulting in significantly less duration spent in the light box (P = 0.003) and noticeably longer time in the dark box (P = 0.008). However, the animals treated with DEMG alone at 50 and 100 mg/kg/day showed increased exploration time of the light zone (P < 0.0001) and reduced duration of stay in the darker compartment (P < 0.0001) showing that DEMG had the potential to yield anxiolytic effects in mice. Furthermore, in comparison to the AlCl_3_-treated control group, the administration of DEMG at 50 and 100 resulted in anxiolytic-like behavior in mice as these mice had longer visits of the light zone with P = 0.005 (DEMG 50+AlCl_3_), and P = 0.0003 (DEMG 100+AlCl_3_) (Fig. 4A), respectively. Further, the increased preference for light areas by DEMG eventually resulted in a reduced duration of stay in the dark zone of the L/D box with P < 0.0001, showing that DEMG at both doses protected the AlCl_3_-induced anxiogenic effects noted in AlCl_3_-treated control (Fig. 4B).

### 3.4 Hole board test

**Fig. 5:**
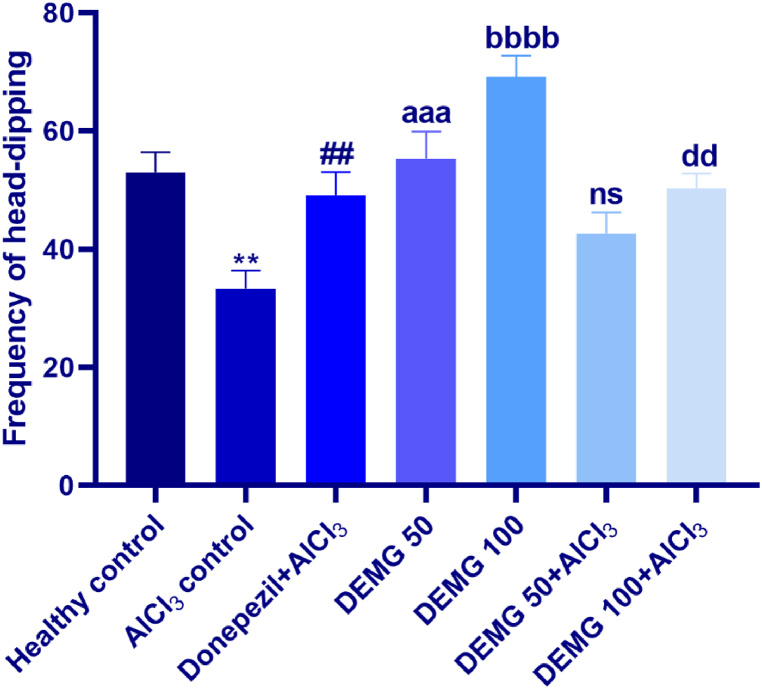
The impact of Demaghi on anxiety-related activity in hole board test. After treatments with DEMG alone and with simultaneous administration of AlCl_3_, the anxiety-like behavior was evaluated by monitoring the mice for 5 min in the maze to note the frequency of head dipping. All data were described in mean ± S.E.M (n = 6) while statistical analysis was performed by one-way ANOVA preceded by Dunnett Test comparing all groups with AlCl_3_ control. **P˂0.01 comparison among healthy control and AlCl_3_ control, ^##^P˂0.01 comparison between Donepezil + AlCl_3_ and AlCl_3_ control, ^aaa^P˂0.001 comparison among DEMG 50 and AlCl_3_ control, ^bbbb^P˂0.0001 comparison among DEMG 100 mg and AlCl_3_ control, ^dd^P˂0.01 comparison between DMG 100+AlCl_3_ and AlCl_3_ control.

This test was also conducted to determine the possible anxiolytic-like impact of DEMG on an anxiogenic arena of holes-comprising square-shaped arena board. The one-way ANOVA demonstrated a statistically substantial variation with all experimental groups in terms of frequency of head-dipping [F (6,34) = 9.701, P < 0.0001]. In detail, the animals of the healthy group showed increased head dips which were decreased in AlCl_3_-treated control with P = 0.002 revealing that administration of AlCl_3_ resulted in anxiogenic effects in mice. The animals given DEMG alone at 50 and 100 mg/kg/day had significantly less anxiety-like behavior towards the hole as they displayed a higher frequency of head dipping with P = 0.0006 and P < 0.0001, respectively. Furthermore, at DEMG 100 exerted neuroprotection effectively as administration of AlCl_3_ could not precipitate the fear towards anxiogenic holes in the animals of this group and they freely explored the holes more often with P = 0.009, in comparison to the AlCl_3_-treated control group as depicted in Fig. 5.

### 3.5 Marble burying test

**Fig. 6:**
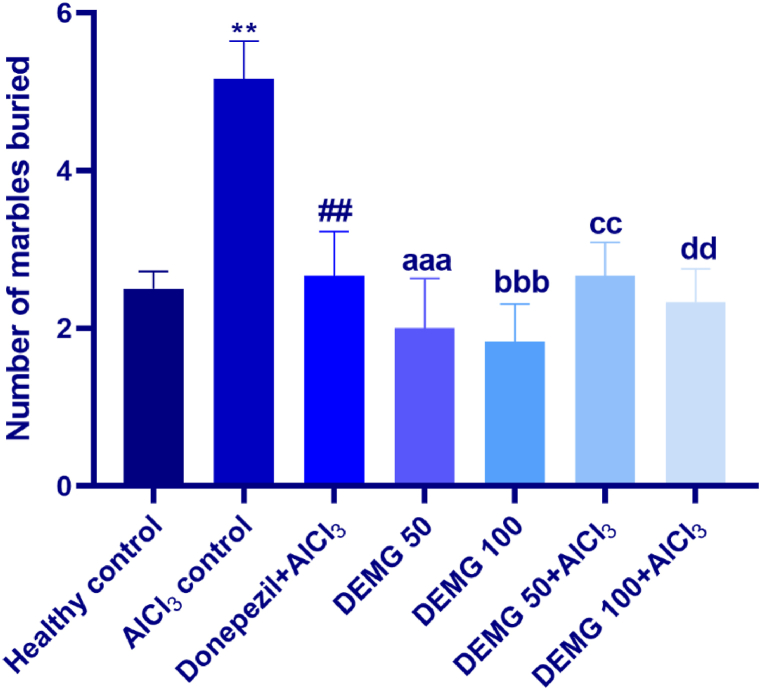
The impact of Demaghi on anxiety-related activity in marble bury test. After treatments with DEMG alone and with simultaneous administration of AlCl_3_, the anxiety-like behavior was evaluated by monitoring the mice for the number of marbles buried. All data were described in mean ± S.E.M (n = 6) while statistical analysis was performed by one-way ANOVA preceded by Dunnett Test comparing all groups with AlCl_3_ control. **P˂0.01 comparison among healthy control and AlCl_3_ control, ^##^P˂0.01 comparison between Donepezil + AlCl_3_ and AlCl_3_ control, ^aaa^P˂0.001 comparison between DEMG 50 and AlCl_3_ control, ^bbbb^P˂0.0001 comparison among DEMG 100 and AlCl_3_ control, ^cc^P˂0.01 comparison between DEMG 50+AlCl_3_ and AlCl_3_ control, ^dd^P˂0.01 comparison between DEMG 100+AlCl_3_ and AlCl_3_ control.

In this test, the animals of different groups varied in digging behavior as the number of buried marbles was notably different among all groups [F (6,35) = 9.70, P < 0.0001]. In detail, the healthy animals showed a significantly reduced several buried marbles (P = 0.002) compared to the AlCl_3_-treated control revealing that administration of AlCl_3_ resulted in increased anxiety-like behavior in mice. However, the treatments of DEMG at doses of 50 and 100 mg/kg/day brought significant anxiolytic effects in mice as these animals buried significantly fewer marbles with P = 0.0006 and P < 0.0001, compared to the AlCl_3_-treated control. Furthermore, the co-administration of DEMG at the dose of 100 with AlCl_3_ significantly protected from AlCl_3_-induced anxiogenic behavior in mice with P = 0.009 revealing that DEMG had the potential to protect the mice brain from the deteriorative effects of AlCl_3_ (Fig. 6).

### 3.6 Y-maze test

**Fig. 7:**
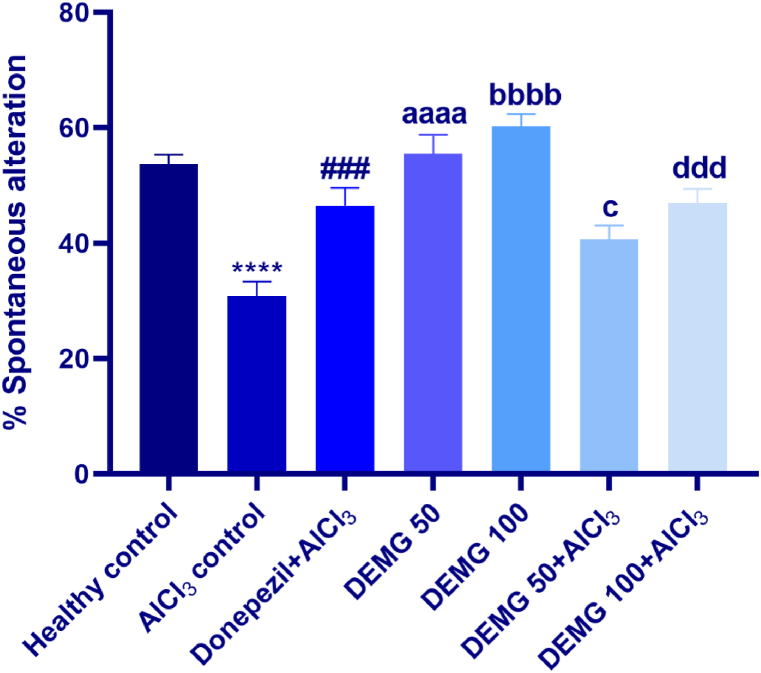
The impact of Demaghi on short-term cognitive ability in AlCl_3_-amnesic mice in Y-maze test. After treatments with DEMG alone and with simultaneous administration of AlCl_3_, the short-term memory was evaluated by monitoring the mice for % spontaneous alteration behavior. All data were described in mean ± S.E.M (n = 6) while statistical analysis was performed by one-way ANOVA preceded by Dunnett Test comparing all groups with AlCl_3_ control. ****P˂0.0001 comparison of healthy control with AlCl_3_ control, ^###^P˂0.001 comparison among Donepezil + AlCl_3_ and AlCl_3_ control, ^aaaa^P˂0.0001 comparison between DEMG 50 and AlCl_3_ control, ^bbbb^P˂0.0001 comparison between DEMG 100 and AlCl_3_ control, ^c^P˂0.05 comparison between DEMG 50+AlCl_3_ and AlCl_3_ control, ^ddd^P˂0.001 comparison between DEMG 100+AlCl_3_ and AlCl_3_ control.

In this test, by calculating the spontaneous alteration in 5 min, the effects of DEMG on the short-term memory of the animals were noted to be significantly different among all groups [F (6,35) = 14.93, P < 0.0001]. Compared to healthy animals, AlCl_3_-amnesic control preferred to investigate the previously visited arms more often, indicating that they had poor recalling capacity and remembrance of the recently explored arm resulting in significantly reduced (P < 0.0001) alternation behavior. But animals receiving DEMG alone at both doses tend to visit unexplored arm more often demonstrating significantly increased spontaneous alternation (P < 0.0001) as shown Fig. 7. Moreover, DEMG also protected the mice from the amnesic effects of AlCl_3_ as these animals had notably increased cognitive ability to recall the recently visited arm and preferred the other arm to enter leading to increased alternation behavior with P = 0.048 (DEMG 50+AlCl_3_) and P = 0.0005 (DEMG 100+AlCl_3_).

### 3.7 Passive avoidance test

**Fig. 8:**
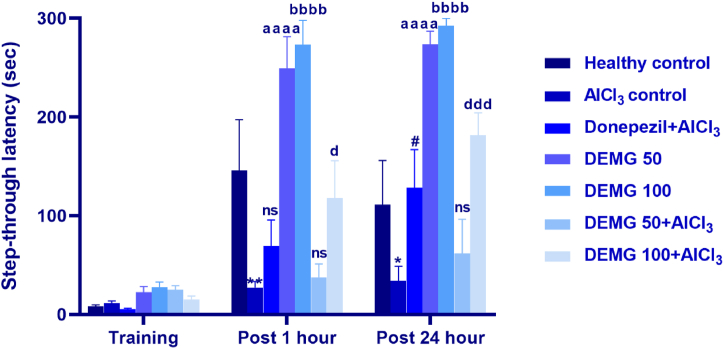
The impact of Demaghi on learning and memory in AlCl_3_-amnesic mice in passive avoidance test. After treatments with DEMG alone and with simultaneous administration of AlCl_3_, mice's learning and memory were tested in post 1 and post 24 h trial carried out after training session to note their step-through latencies. All data were described in mean ± S.E.M (n = 6) while statistical analysis was performed by two-way ANOVA preceded by Dunnett Test comparing all groups with AlCl_3_ control. *P˂0.05, **P˂0.01 comparison between healthy control and AlCl_3_ control, ^#^P˂0.005 comparison between Donepezil + AlCl_3_ and AlCl_3_ treated group, ^aaaa^P˂0.0001 comparison among DEMG 50 and AlCl_3_ control, ^bbbb^P˂0.0001 comparison between DEMG 100 and AlCl_3_ control, ^d^P˂0.005, ^ddd^P˂0.001 comparison between DEMG 100+AlCl_3_ and AlCl_3_ control while ns shows non-significant outcomes.

The passive avoidance test evaluated the effects of DEMG on long-term remembrance in mice by testing them for learning and memory retention after 1 h and 24 h of aversive stimuli applied during the test trial. The outcomes of two-way ANOVA revealed that step-through latency varied significantly between all experimental groups [F (6,84) = 25.50, P < 0.0001]. In detail, as compared to healthy mice, the AlCl_3_-treated amnesic control showed reduced latencies to visit the dark compartment with P = 0.0018 (post 1 h) and P = 0.04 (post 24 h), indicating their poor remembrance of shock applied during the training session. But the animals treated with DEMG at 50 and 100 mg/kg/day had better memory as their step-through latencies were significantly longer (P < 0.0001) in both test trials (Fig. 8). Further, the animals simultaneously treated with DEMG 100 and AlCl_3_ also had significantly increased step-through latencies after 1 h (P = 0.04) and 24 h (P = 0.0002), revealing that chronic intake of DEMG protects from memory impairment in mice.

### 3.8 Water maze test

**Fig. 9:**
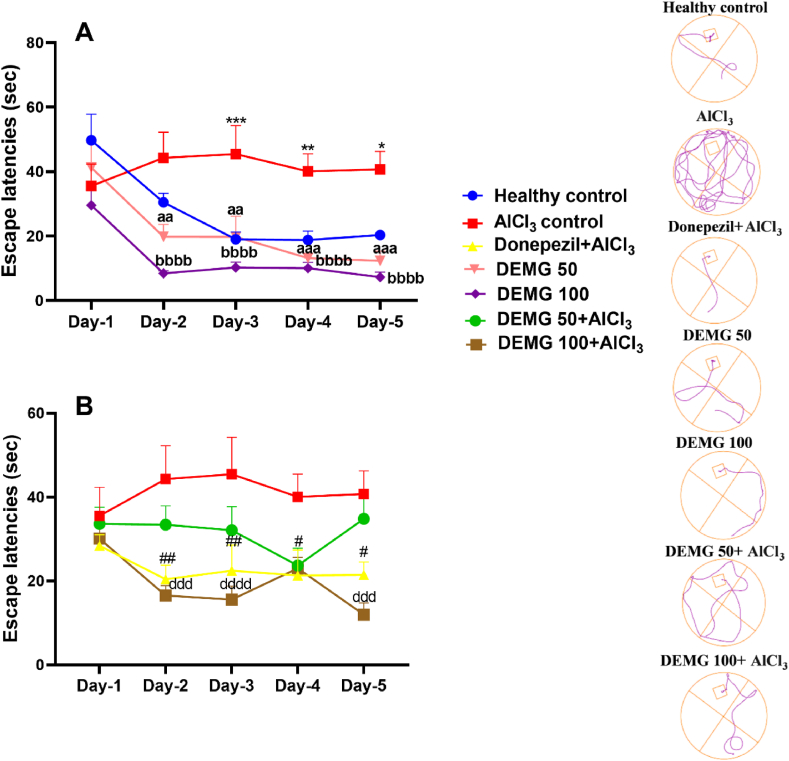
The impact of Demaghi on learning and memory in AlCl_3_-amnesic mice in the Morris water maze test. After treatments with DEMG alone and with simultaneous administration of AlCl_3_, the learning and memory of mice were tested for 120 s in training and test trials conducted on consecutive 5 days to express the escape latencies of (A) healthy control, AlCl_3_ control, DEMG 50 and DEMG 100 and (B) AlCl_3_ control, donepezil, DEMG 50+AlCl_3_ and DEMG 100+AlCl_3_ with representative track plots of all groups. All data were described in mean ± S.E.M (n = 6) while statistical analysis was performed by two-way ANOVA preceded by Dunnett Test comparing all groups with AlCl_3_ control. *P˂0.05, **P˂0.01, ****P˂0.0001 comparison among healthy control and AlCl_3_ control, ^##^P˂0.01, ^####^P˂0.0001 comparison among Donepezil + AlCl_3_ and AlCl_3_ control, ^aa^P˂0.01 ^aaaa^P˂0.0001 comparison between DEMG 50 and AlCl_3_ control, ^bbb^P˂0.001, ^bbbb^P˂0.0001 comparison among DEMG 100 and AlCl_3_ control, ^cccc^P˂0.0001 comparison between DEMG 50+AlCl_3_ and AlCl_3_ control, ^ddd^P˂0.001, ^dddd^P˂0.0001 comparison between DEMG 100 and AlCl_3_ control.

The two-way ANOVA showed a significant variation of escape latencies among the groups during five days [F (6,140) = 18.81, P < 0.0001]. Despite the initial two training days, the AlCl_3_-amnesic animals continued to exhibit significantly longer escape latencies on day 3 (P = 0.002), which was further increased on day 5 (P < 0.0001), in comparison to the healthy control group demonstrating their inability to recall the location of the platform. However, compared to amnesic control, the animals administered with DEMG (50 and 100 mg/kg/day) effortlessly took less time to find the submerged platform as their escape latencies were noticeably shorter with P < 0.0001 on day 5 (Fig. 9A). The DEMG at both doses co-administered with AlCl_3_ animals showed significant protection from the deteriorative effects of AlCl_3_ on animal memory. These mice recognized the platform's location very quickly, resulting in their notably shorter escape latencies (P < 0.0001) compared to AlCl_3_-amnesic control (Fig. 9B).

**Fig. 10:**
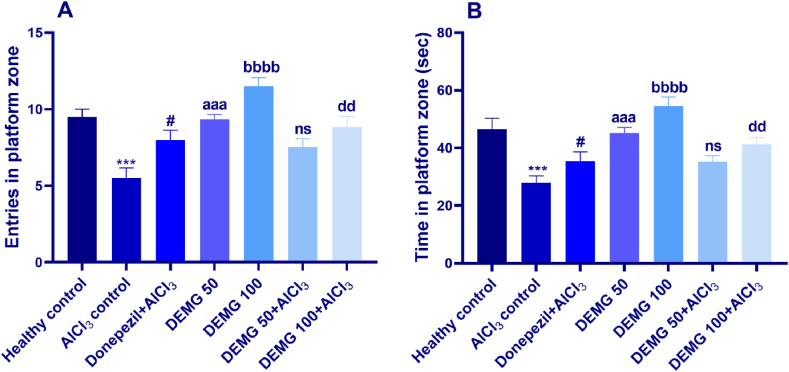
The impact of Demaghi on memory retention in AlCl_3_-amnesic mice on probe day in Morris water maze test. After treatments with DEMG alone and with simultaneous administration of AlCl_3_, the memory of mice was tested for 120 s in probe trial carried out on day 6 of the MWM test to note (A) entries in platform region and (B) time in platform region. All data were described in mean ± S.E.M (n = 6) while statistical analysis was performed by two-way ANOVA preceded by Dunnett Test comparing all groups with AlCl_3_ control. ***P˂0.001 comparison between healthy control and AlCl_3_ control, ^#^P˂0.05 comparison between Donepezil + AlCl_3_ and AlCl_3_ control, ^aaa^P˂0.001 comparison among DEMG 50 and AlCl_3_ control, ^bbbb^P˂0.0001 comparison between DEMG 100 and AlCl_3_ control, ^dd^P˂0.01 comparison between DEMG 100+AlCl_3_ and AlCl_3_ control while ns shows non-significant outcomes.

On day 6 of the experiment, the platform was removed. Animals were tested for memory retention of the platform quadrant by monitoring their entries and swimming time in the quadrant where the platform was previously situated. The statistical analysis through one-way ANOVA revealed a significant inter-group variation for the number of entries [F (6,35) = 10.47, P < 0.0001] and time spent in the platform zone [F (6,35) = 10.11, P < 0.0001]. The AlCl_3_-treated amnesic mice displayed poor recalling capacity of the rescuing quadrant as they entered that zone less frequently (P = 0.0001) and swam there for shorter periods (P = 0.0002) than healthy animals. The animals treated with DEMG at both doses (50 and 100 mg/kg/day) resulted in dose-dependent improvement in observed parameters as DEMG 50 animals had significantly increased entries (P = 0.0002) and spent longer duration there (P = 0.0006). Moreover, both parameters were further improved in animals treated with DEMG 100 as the entries and duration spent in the target quadrant were noticeably increased (P < 0.0001) compared to amnesic mice. Additionally, the treatment of animals with DEMG 100 and simultaneous administration of AlCl_3_ resulted in the protection of mice from memory impairment. These animals showed better remembrance of the target zone as they entered the zone significantly more often (P = 0.0014) and spent more time there (P = 0.0081) compared to the amnesic group. As shown in Fig. 10A and B. However, the outcomes remained unchanged in animals treated with DEMG 50 and AlCl_3_.

### 3.9 Sucrose preference test

**Fig. 11:**
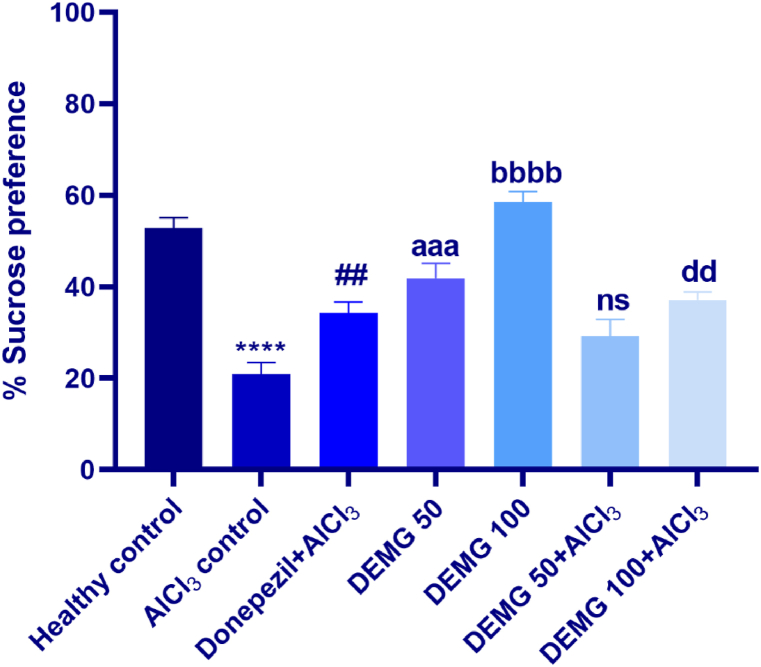
The impact of Demaghi on depression-like behavior of mice in the sucrose preference test. After treatments with DEMG alone and with simultaneous administration of AlCl_3_, the depression-like behavior was assessed by monitoring the mice for % sucrose preference. All data were described in mean ± S.E.M (n = 6) while statistical analysis was performed by two-way ANOVA preceded by Dunnett Test comparing all groups with AlCl_3_ control. ****P˂0.0001 comparison among healthy control and AlCl_3_ control, ^##^P˂0.01 comparison among Donepezil + AlCl_3_ and AlCl_3_ control, ^aaa^P˂0.001 comparison between DEMG 50 and AlCl_3_ control, ^bbbb^P˂0.0001 comparison between DEMG 100 and AlCl_3_ control, ^dd^P˂0.01 comparison among DEMG 100+AlCl_3_ and AlCl_3_ control while ns shows non-significant outcomes.

The sucrose preference test showed that animals of differently treated groups had different anhedonia and depression-like behavior [F (6,35) = 23.93, P < 0.0001]. Compared to the healthy mice, the statistical evaluation showed a significant reduction in sucrose consumption in the AlCl_3_ with P < 0.0001 showing considerable melancholy in these animals, indicating depressive-like behavior. Animals provided with DEMG 50 and 100 consumed more sucrose, with P = 0.0004 and P < 0.0001, respectively. Moreover, as compared to AlCl_3_ control mice, the animals co-administered with DEMG 100 and AlCl_3_ had protection from depression-like behavior as they consumed significantly more sucrose (P < 0.0001) revealing the protection from the neurotoxic effects of AlCl_3_ on the brain (Fig. 11).

### 3.10 Biochemical analysis

**Fig. 12:**
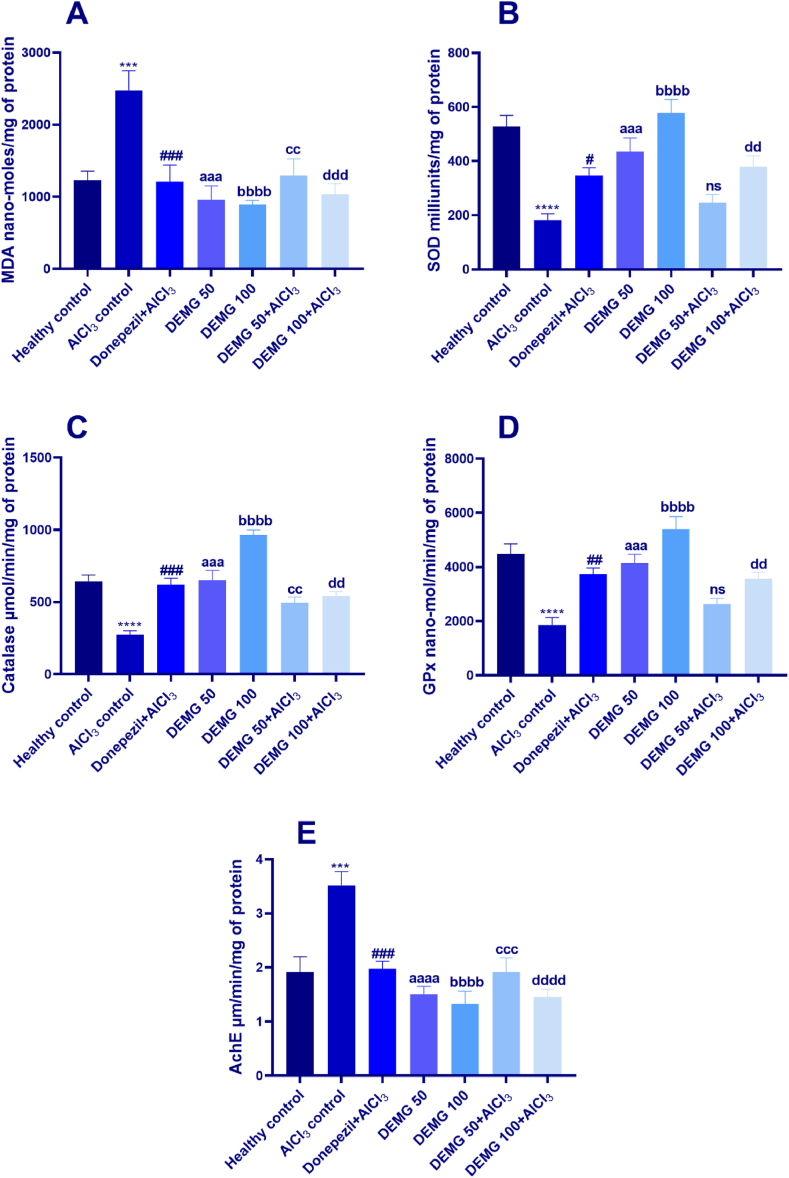
The impact of Demaghi on the activity of (A) MDA, (B) SOD, (C) Catalase, (D) GPx and (E) AchE in an isolated mice brain. All data were described in mean ± S.E.M (n = 4) while statistical analysis was performed by one-way ANOVA preceded by Dunnett Test comparing all groups with AlCl_3_ control. ***P˂0.001, ****P˂0.0001 comparison among healthy control and AlCl_3_ control, ^#^P˂0.05, ^##^P˂0.01, ^###^P˂0.001 comparison between Donepezil + AlCl_3_ and AlCl_3_ control, ^aaa^P˂0.001, ^aaaa^P˂0.0001 comparison among DEMG 50 and AlCl_3_ control, ^bbbb^P˂0.0001 comparison between DEMG 100 and AlCl_3_ control, ^cc^P˂0.01, ^ccc^P˂0.001 comparison between DEMG 50+AlCl_3_ and AlCl_3_ control, ^dd^P˂0.01, ^ddd^P˂0.001, ^dddd^P˂0.0001 comparison between DEMG 100+AlCl_3_ and AlCl_3_ control while ns shows non-significant outcomes.

The statistical analysis revealed substantial variations among each group with [F (6, 21) = 7.83, P = 0.0002]. In detail, the animals of AlCl_3_-treated control had increased brain oxidative stress as MDA levels were significantly higher (P = 0.0009) compared to healthy animals. Treating mice with DEMG 50 and 100 resulted in significant protection from lipid peroxidation with P < 0.0001. Moreover, DEMG at both doses protected the mice brain from AlCl_3_-induced oxidative stress and lipid peroxidation as mice simultaneously administered with DEMG and AlCl_3_ had significantly reduced MDA levels with P = 0.001 (DEMG 50+AlCl_3_) and P = 0.0002 (DEMG 100+AlCl_3_), in comparison to AlCl_3_-treated control (Fig. 12A).

The isolated brains of animals were also tested for SOD levels which were noted to be significantly different among all groups [F (6,21) = 13.34, P < 0.0001]. The AlCl_3_-treated mice had the lowest SOD activity (P < 0.0001) in comparison to the healthy group. The four week administration of DEMG at 50 and 100 mg/kg/day resulted in increased activity of SOD enzyme with P = 0.0009 and P < 0.0001, respectively (Fig. 12B). Moreover, DEMG at a dose of 100 mg/kg/day protected the mice brains from the deteriorative effects of AlCl_3_ as mice co-administered with DEMG 100 and AlCl_3_ showed noticeably increased SOD levels (P = 0.009), in comparison to AlCl_3_-treated amnesic control.

Additionally, the outcomes of one-way ANOVA showed that catalase levels had significant variations across all treatments [F (6,21) = 23.83, P < 0.0001]. The post hoc test revealed that in comparison to healthy animals, catalase levels were notably reduced in AlCl_3_-treated amnesic control with P < 0.0001. However, the chronic intake of DEMG 50 and 100 mg/kg/day resulted in significantly elevated catalase levels with P < 0.0001, compared to the AlCl_3_-treated amnesic animals (Fig. 12C). Further, the AlCl_3_-induced deterioration was protected in animals treated by DEMG at both doses as SOD levels were increased with P = 0.007 (DEMG 50+AlCl_3_) and P = 0.001 (DEMG 100+AlCl_3_).

Similarly, the statistical analysis demonstrated significant variations in GPx levels among all groups [F (6,21) = 13.75, P < 0.0001]. The mice treated with AlCl_3_ showed a pronounced reduction in GPx levels (P < 0.0001), compared to healthy animals. But the treatment of mice with DEMG 50 and 100 mg/kg/day resulted in a dose-dependent increase in GPx levels with P = 0.0003 and P < 0.0001, respectively. Moreover, a dose of 100 mg/kg/day also protected the mice from AlCl3-induced decline in antioxidant defenses as GPX levels were significantly increased (P = 0.005), compared to amnesic animals (Fig. 12D).

In addition to the antioxidant enzymes, the activity of acetylchoninesterase enzyme (AchE) were also noted in isolated brains which significantly varied among all groups [F (6,21) = 11.57, P < 0.0001]. The treatment of mice with AlCl_3_ caused a significant elevation in AchE activity (P = 0.0002) compared to the healthy control group. But, the intake of DEMG 50 and 100 mg/kg/day lowered AchE potential with P < 0.0001, revealing this herbal tonic might have phytoconstituents with cholinergic potential (Fig. 12E). Further, the AlCl_3_-induced elevation of AchE levels was also significantly reduced by DEMG at both doses with P = 0.0002 (DEMG 50+AlCl_3_) and P < 0.0001 (DEMG 100+AlCl_3_), compared to AlCl_3_-induced amnesic animals.

### 3.11 Histopathological analysis

**Fig. 13:**
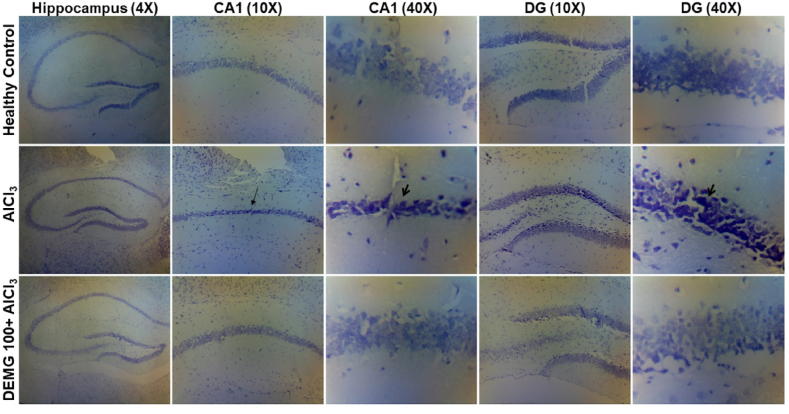
The impact of Demaghi in preventing Aluminum Chloride induced toxicity. Nissl staining of isolated brain tissue from healthy control mice (top panel), mice treated with AlCl_3_ (middle panel) and mice co-administered with DEMG 100 + AlCl_3_ (bottom panel) indicating hippocampal CA1 and DG region at 10× and 40× magnification.

Histopathological analysis of the neuronal tissue through nissl staining revealed that administration of aluminum chloride induced neurotoxicity in hippocampus region of the brain (Fig. 13). The damage to the neuronal tissue was evident by the thinning of pyramidal layer in CA1 region of mice treated with aluminum chloride (indicated by long arrow). The neuronal bodies in CA1 and DG regions also appeared shrunk and darkly stained, showing signs of degeneration (indicated by arrow heads). These abnormalities were prevented in mice with co-administration of DEMG with AlCl_3_ where the neuronal bodies appeared intact and showed lesser signs of shrinkage or vacuolation in the tissues.

### 3.12 Chemical characterization

**Fig. 14:**
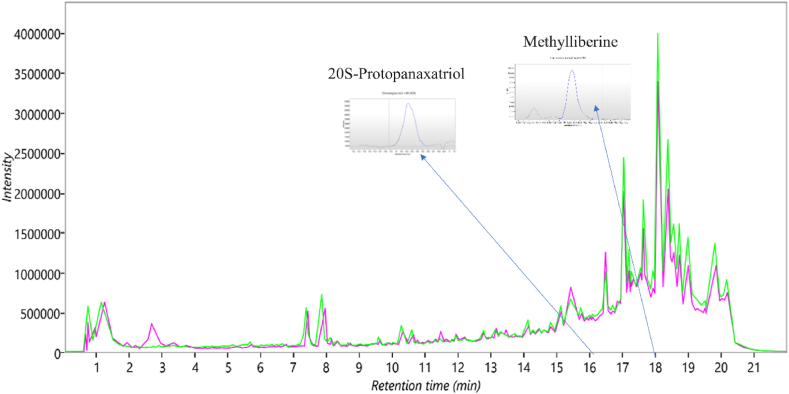
Total Ion Chromatogram (TIC) of DEMG extract in positive mode.

The chemical characterization of the Demaghi was done by UPLC-MS analysis to identify the key phytoconstituents present in this herbal formulation. Through UPLC-MS analysis a total of 19,507 mass ion features were detected in positive ionization mode (Fig. 14). After applying the filter using ANOVA p-value ≤0.05, the remaining features were reduced to 10,898. Among these, 6924 features were the highest in the sample compared to the background. These features were screened in different databases to identify the phytoconstitutents. Out of 6924 features only 1008 were identified using the NIST database, NIST spectra, and NIST chemistry WebBook while 204 features were identified using the PlantCyc database, Planta Piloto de Química Fina. Universidad de Alcalá database (Detailed list containing the name of compounds, mass to charge ratio *m*/*z*, retention time, p & q values, and abundance of compound is shared in the supplementary data). The identified phytoconstituents beloneged to various classes and it included compounds (e.g. quercitin, gypsogenic acid) which are naturally occurring in many plant species while there were some compounds (e.g. neoisoliquiritin, cinnamyl acetate) that are limited to only to a specific family of plants. Few of the identified compounds included hederagenin, 20S-protopanaxatriol, enoxolone, cycloheximide, quercetin, neoisoliquiritin, curcumin monoglucoside, vernolic acid, *cis*-(−), methylliberine, (E)-cinnamyl acetate, methoxsalen, 4-hydroxyphenyllactic acid and citicoline have been previously reported in the literature to modulate cholinergic signaling and posses neuropharmacological properties.

### 3.13 Molecular docking

**Fig. 15:**
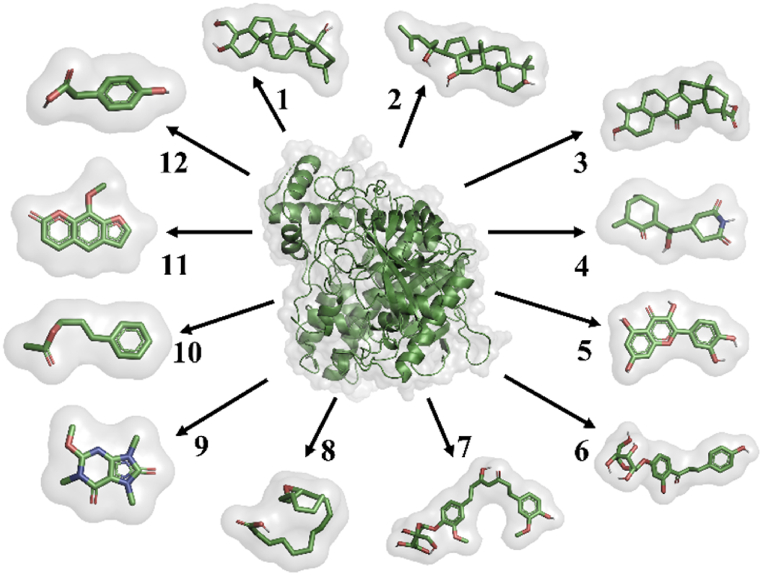
Molecular docked targeted ligands with acetylcholinesterase 4EY7.

As anticholinesterase property of the herbal formulation was revealed in the biochemical analysis, the identified phytoconstituents, hederagenin, 20S-protopanaxatriol, enoxolone, cycloheximide, quercetin, neoisoliquiritin, curcumin monoglucoside, vernolic acid *cis*-(−), methylliberine, (E)-cinnamyl acetate, methoxsalen and 4-hydroxyphenyllactic acid were tested *in-silico* for their potential to inhibit acetylcholinesterase enzyme, to boost cholinergic signalling (Fig. 15). As results given in Table 1, the investigated ligands (named 1 to 12 in their respective sequence) have varying degrees of Glide score, hydrogen bonding, hydrophobic interactions, and electrostatic interactions with the acetylcholinesterase (AChE), a protein having PDB ID 4EY7.

**Table 1:** Glide score, Hydrogen binding, hydrophobic and electrostatic interactions along with distances (in Å) for investigated ligands using acetylcholinesterase protein having PDB ID 4EY7.

Ligands with 4E7Y	Glide Gscore	Hydrogen-Binding Interaction, Residue (Distance Å)	Hydrophobic Interaction, Residue (Distance Å)	Electrostatic Interaction, Residue (Distance Å)
1 Hederagenin	−8.7	ASN533(2.00) H–O (2.00) TRP532(2.94)		
2 20S-Protopanaxatriol	−8.9	TYR341(2.29) TYR341(2.13) H–O (2.30)	TYR124(4.45)	PHE297(5.37) PHE338(5.23) TYR341(5.33)
3 Enoxolone	−10.0	ARG296(2.31) HIS405(2.17) GLN369(2.72)		
4 Cycloheximide	−7.6	SER293(1.97) H–O (2.33)	HIS287(3.61)	
5 Quercetin	−8.5	TYR124(2.11)	PHE338(4.98)	TYR124(5.17) TYR124(5.68) TYR341(5.55)
6 Neoisoliquiritin	−8.2	ARG296(2.36) ARG296(2.74) H–O (2.18) GLN369(2.09) H–O (2.48) H–O (1.75) GLN369(2.15)	PRO235(3.61) PRO235(4.95) PRO410(4.39)	
7 Curcumin monoglucoside	−7.5	TYR124(2.58) H–O (2.27) H–O (2.45) LEU289(1.83)	TRP286(3.77) SER293(3.61) ARG296(3.70) SER293(3.71) TRP286(4.19)	TRP286 (5.76) TRP286(5.11)
8 Vernolic acid	−4.3	ASN233(2.03)	HIS405(4.67)	PRO235(5.42
9 Methylliberine	−6.1	TYR124(2.39)	VAL294(3.68) TYR124(3.29) SER293(3.71) TRP286(3.62) TRP286(4.30) TYR341(4.32)	TRP286(5.93)
10 Cinnamyl acetate	−7.4		TYR341(4.21)	PHE297(5.84)
11 Methoxsalen	−8.6	GLY121(2.60) HIS447(2.73) TYR124(2.70)	GLU202(3.22) HIS447(4.36) HIS447(3.81) TYR341(3.96) PHE338(4.68) HIS447(4.72) PHE295(4.99) PHE297(4.62) HIS447 (4.67)	TRP86(5.53) TYR337(5.43) TYR341(5.29) HIS447 (5.15) TRP86(5.31) PHE338(5.10)
12 4-Hydroxyphenyllactic acid	−6.5	GLY121(2.51) GLY122(2.40)		PHE338(5.04)
Donepezil	−11.1	PHE295(2.02)	TRP286(3.62) TRP286(3.94) TRP86(4.37) TRP86(4.20) TYR341(4.03) GLY121(4.18) TYR72(4.65) TRP286(4.85) PHE338(4.66)	GLY120(5.44) TRP286(5.42) LEU289(5.31)

The Glide score values for the ligands range from −10.0 kcal/mol (ligand 3 enoxolone) to −4.3 kcal/mol (ligand 8 vernolic acid), indicating that ligand 3 has the strongest Glide score and ligand 8 has the weakest Glide score. Ligands 2, 5, 6, and 11 also have relatively strong binding affinities, with ΔG values of −8.9, −8.5, −8.2, and −8.6 kcal/mol, respectively. Hydrogen bonding interactions are crucial for ligand binding to the protein and were analyzed for each ligand Table 2. Ligand 1 (hederagenin) shows hydrogen bonding interactions with the protein residue ASN533 (2.00 Å) and TRP532 (2.94 Å). Ligand 2 (20S-protopanaxatriol) forms hydrogen bonds with TYR341 (2.29 Å and 2.13 Å) and H–O (2.30 Å). Ligand 3 (enoxolone) interacts with ARG296 (2.31 Å), HIS405 (2.17 Å), and GLN369 (2.72 Å) through hydrogen bonding. Ligand 4 (cycloheximide) forms a hydrogen bond with SER293 (1.97 Å) and H–O (2.33 Å). Ligand 5 (quercetin) forms hydrogen bonds with TYR124 (2.11 Å and 5.17 Å) and TYR341 (5.55 Å). Ligand 6 (neoisoliquiritin) forms hydrogen bonds with ARG296 (2.36 Å and 2.74 Å), H–O (2.18 Å), GLN369 (2.09 Å), H–O (2.48 Å), and H–O (1.75 Å). Ligand 7 (curcumin monoglucoside) shows hydrogen bonding interactions with TYR124 (2.58 Å), H–O (2.27 Å), H–O (2.45 Å), LEU289 (1.83 Å), TRP286 (3.77 Å), SER293 (3.61 Å), ARG296 (3.70 Å), SER293 (3.71 Å), and TRP286 (4.19 Å). Ligand 8 (vernolic acid) indicates hydrogen bonding with ASN233 (2.03 Å). Ligand 9 (methylliberine) interacts with TYR124 (2.39 Å), VAL294 (3.68 Å), SER293 (3.71 Å), TRP286 (3.62 Å), TRP286 (4.30 Å), and TYR341 (4.32 Å) through hydrogen bonding. Ligand 10 (cinnamyl acetate) forms a hydrogen bond with TYR341 (4.21 Å). Ligand 11 (methoxsalen) shows hydrogen bonding interactions with GLY121 (2.60 Å), HIS447 (2.73 Å), TYR124 (2.70 Å), GLU202 (3.22 Å), HIS447 (4.36 Å), HIS447 (3.81 Å), TYR341 (3.96 Å), PHE338 (4.68 Å), HIS447 (4.72 Å), PHE295 (4.99 Å), and PHE297 (4.62 Å). Lastly, ligand 12 (4-hydroxyphenyllactic acid) interacts with GLY121 (2.51 Å) and GLY122 (2.40 Å) through hydrogen bonding. The native crystal structure of the 4EY7 protein shows how donepezil binds to AChE at its active site. We also used donepezil for cross-validation molecular docking purposes and results showed that donepezil, as compared to other investigated ligands, has the strongest Glide score (−11.1 kcal/mol), fits snugly into the enzyme's active site, and forms multiple hydrogen bonds and hydrophobic interactions, stabilizing the complex. This binding interaction prevents the enzyme from breaking down acetylcholine, leading to an accumulation of the neurotransmitter in the brain.

**Table 2:**
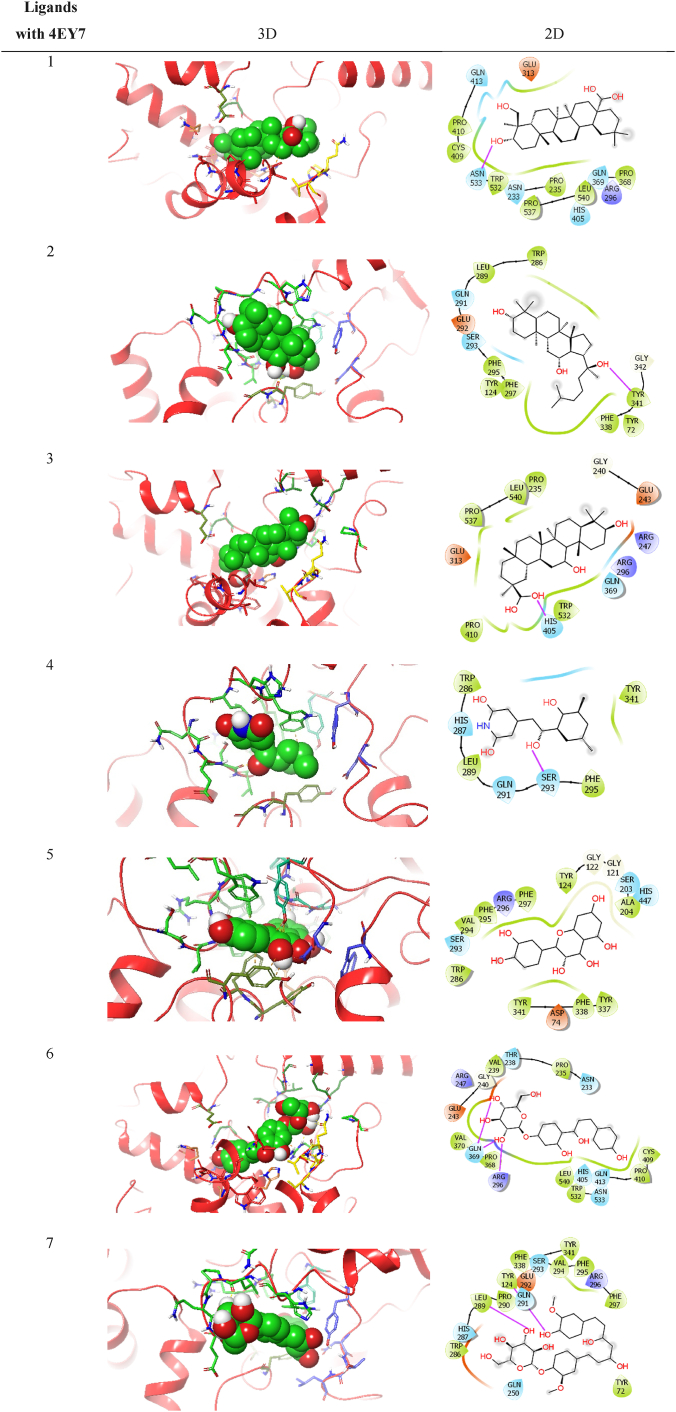

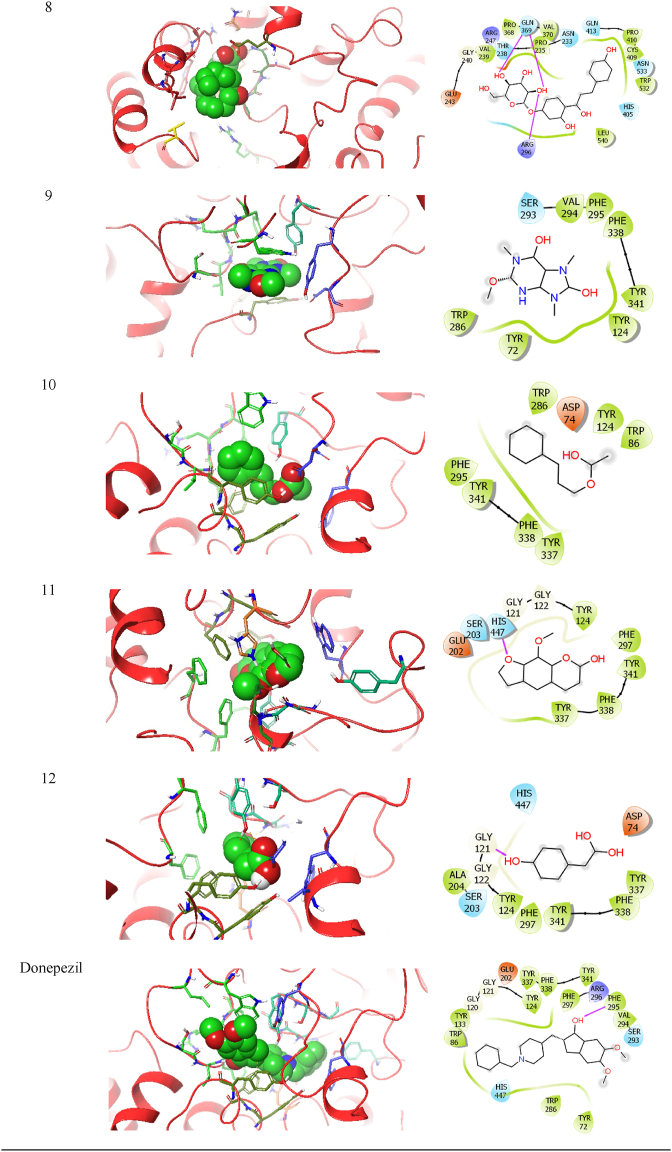
Ligand interaction diagram for all the investigated ligands (1–12) and standard drug donepezil with acetylcholinesterase protein 4EY7.

In conclusion, the Glide score and interactions of the investigated ligands with acetylcholinesterase provide important insights into their potential as inhibitors of this enzyme. These findings could have significant implications for developing new drugs for treating diseases like Alzheimer's. However, further studies, such as in vitro and in vivo experiments, would be required to validate these results and to explore the therapeutic potential of these ligands. Additionally, structure-activity relationship studies could be performed to further optimize the Glide score and selectivity of these ligands. Overall, these results could pave the way for the discovery of new and effective treatments for acetylcholinesterase-related diseases.

## 4 Discussion

AD is a neurodegenerative disorder commonly associated with advanced age. The unavailability of adequate medicinal treatment has made this disorder a serious concern for global health management [37]. The progression of this disease results in behavioral changes and cognitive deficits contributing as the most common cause of dementia worldwide [38]. Cholinesterase inhibitors are commonly prescribed medication for AD, which cause symptomatic improvements by increasing the levels of acetylcholine [39]. With the increase in age expectancy and rise in the older population throughout the world, it is important to look forward to new neuroprotective compounds capable of crossing the blood-brain barrier to counter the pathological events involved in the onset and progression of neurodegenerative disorders.

Some reported mechanisms through which aluminum causes neurotoxicity are increased oxidative stress and impaired cholinergic function [5]. Its higher doses and prolonged exposure exerts neurodegenerative effects on the cerebral cortex and hippocampus, brain regions involved in memory and learning skills [40]. After a comprehensive literature analysis, it is the first study to evaluate the neuroprotective effects of the herbal product, Demaghi, against aluminium chloride-induced neurotoxicity in mice.

In the present study, the exposure of mice to aluminium chloride exerted a negative impact on anxiety-like behavior, which is following previous findings of Buraimoh et al. [41]. However, the therapeutic components of Demaghi protected mice from the anxiogenic effects of aluminium chloride as DEMG-treated mice fearlessly explored open, exposed and illuminated zone in the OFT, EPM and L/D test. Further, DEMG-treated mice managed to explore the anxiogenic holes and marbles in HBT and MBT tests, respectively. GABA is the main inhibitory neurotransmitter in the brain and most anxiolytic drugs exert anxiolytic effects by facilitating GABAergic action. Quercetin and curcumin present in DEMG are well-known GABA-modulating phytocompounds and their long-term administration might be yielding observed anxiolytic outcomes [42,43].

The cholinergic hypothesis proposes that dysfunction of cholinergic neurons and a decrease in acetylcholine works primarily in precipitating cognitive decline. Acetylcholinesterase enzyme regulates the synaptic acetylcholine levels as well as this enzyme promotes protein aggregation resulting in neuronal degeneration in AD. Quercetin is known to exhibit anticholinesterase activity as its administration reduced acetylcholinesterase activity in the hippocampus. One of the detected compounds in DEMG was protopanaxatriol which is a ginsenoside and has been reported to improve memory. In a previous study, pre-treatment of mice with protopanaxatriol resulted in improved cholinergic levels and reduced oxidative stress resulting in protection from scopolamine‐induced amnesia [44]. Besides this, hederagenin has also been verified for its cholinesterase-inhibiting potential during in vitro testing, which further attribute to improvement in cognitive deficit by restoring cholinergic systems in the brain [45]. Our results, also showed strong affinity of enoxolone, a pentacyclic triterpenioid with acetylcholinesterase revealing its potential to inhibit this enzyme, thus potentiating cholinergic signalling. The beneficial role of enoxolone in Alzheimer's disease has also been suggested by inhibiting presenilin stabilization factor like protein (PSFL) which plays vital role in beta amyloid plaques formation [46].Overall, the phytocompound with anticholinesterase potential present in DEMG might be modulating the cholinergic system in the brain, resulting in increased learning and memory noted in DEMG-treated mice in Y-maze, PAT, and MWM tests.

Aluminium has been reported to deposit in the cytoplasm and nuclei of AD patients' hippocampal neurons, resulting in the formation of neurofibrillary tangles [47]. The pathogenesis of neurodegenerative diseases, including AD, involves formation of protein aggregates. A combination of phytocompounds capable of reducing the burden of protein aggregation and deposition might prove helpful in preventing neurodegeneration. Hederagenin, phytocompound present in DEMG, has been reported to work actively as an autophagic component of Chinese herbal medicines and facilitates the degradation of neurodegenerative proteins by inducing autophagy resulting in improvement in motor deficits of neurotoxin-injected mice [48]. Quercetin is another phytocompound present in DEMG that has been known to protect neuronal degeneration by modulating the abnormal folding and aggregation of protein [49]. Curcumin also prevents Aβ aggregation by reducing insoluble Aβ concentration [50].

The inadequate antioxidant defense system in the brain causes imbalanced ROS generation and eradication resulting in altered cellular pathways and lipid peroxidation. Aluminum crosses the blood-brain barrier and increases free radical generation leading to impaired mitochondrial functions in different brain regions causing neurodegeneration [51]. Moreover, oxidative stress is also considered as major etiological factor for the progressive decline in cognitive, memory and motor functions in humans and animals [52]. SOD, CAT, and GPx are antioxidant enzymes that were increased after DEMG treatment while MDA, an indicator of lipid peroxidation, was decreased in this study. Quercetin and curcumin actively combat oxidative stress through their radical scavenging and metal-chelating potential [53,54]. Liquiritin is a phytocompound present in DEMG which has been reported to enhance the activity of SOD, CAT and GPx resulting in protection from Aβ-induced oxidative damage [55].

The role of inflammation in the progression of neurodegenerative disorders is evident in the literature. After initial events of neuronal injury, neuroinflammation works as the driving force behind various neuropathologies leading to neuronal degeneration. Curcumin inhibits neuroinflammatory processes as it is a potent inhibitor of the NF-κB signaling pathway resulting in the downregulation of inflammatory mediators [56].

Citicoline is another component of DEMG that has a promising neuroprotective role. Recently reported data suggest that long-term intake of citicoline enhances endogenous neuroprotective mechanisms and slows down the development of neurodegenerative diseases [57]. It plays role in the synthesis of phosphatidylcholine, which is an essential component of the cell membrane, thus stimulating the regeneration of damaged neuronal [58].

Overall, our results suggest a neuroprotective and beneficial role of Demaghi in mice. However, it needs further investigation to ascertain the molecular mechanisms through which it exerts its effects. Moreover, this polyherbal formulation contains many neuropharmacologically active phytoconstitutents as indicated by the UPLC-MS analysis and it is difficult to determine the exact origin of these compounds in the polyherbal formulation. In the next steps, each of the ingredients in formulation can be investigated individually for its contribution in enhancing brain functions and cognition which can be helpful later to devise a more efficient and potent cocktail of neuroprotective compounds.

## 5 Conclusion

The current research supports the neurological benefits of Demaghi, a polyherbal formulation which is commonly used for improving brain function and memory in Pakistan. Its intake protected the mice from neurotoxic effects of aluminium chloride. *In-vivo* behavioral analysis via the OFT, EPM, L/D, hole board, marble burying and sucrose preference tests showed that administration of Demaghi reduced the aluminum induced anxiety and depression in these animals and also improved the memory and cognitive functions in mice as indicated by Y-maze, PAT and MWM tests. Biochemical analysis of brain homogenate also indicated oxidative stress in mice after aluminum exposure which was prevented by the co-administration of Demaghi. These results were further confirmed by histopathological examination of the brain tissue as neuronal loss in hippocampal region was evident in mice treated with aluminum chloride while brain tissue from animals co-administered with Demaghi showed lesser signs of neuronal degeneration. UPLC-MS analysis of this polyherbal formulation identified many potentially active phytoconsitituents which may be responsible for the neuropharmacologial properties. A few of the identified phytoconsitituents were further screened *in-silico* for their potential to inhibit acetylcholinesterase enzyme among which Enoxolone, 20S-Protopanaxatriol and Hederagenin showed higher affinity for the target protein, predicting the mechanism of enhanced cholinergic signalling through this formulation. Overall, this study demonstrates benefits of Demaghi in ameliorating neurotoxin-induced oxidative stress and producing a beneficial impact on cognition by increased cholinergic neurotransmission.

## Author contributions

Hassan Ali: performed the experiments and wrote the paper. Hafiz Usman: performed the experiments, analyzed & curated the data, and wrote the paper. Waseem Ashraf: conceived and designed the experiments, contributed reagents, materials & analysis tools, analyzed & interpreted the data and wrote the paper. Faleh Alqahtani: acquired funding, contributed reagents, materials & analysis tools, analyzed & interpreted the data and wrote the paper. Sana Javaid: analyzed & interpreted the data and wrote the paper. Farhan Siddique: performed the experiments, analyzed & interpreted the data and wrote the paper. Muhammad Fawad Rasool: analyzed & interpreted the data and wrote the paper. Imran Imran: conceived and designed the experiments, contributed reagents, materials & analysis tools, analyzed & interpreted the data and wrote the paper. Tanveer Ahmad: analyzed & interpreted the data and wrote the paper. Anas M. Abdel Rahman: performed the experiments, analyzed & interpreted the data and wrote the paper. Reem H. AlMalki: performed the experiments and wrote the paper.

## Funding

This work was funded by Distinguished Scientist Fellowship program at King Saud University, Riyadh, Saudi Arabia through research supporting project Number (RSP2023R131).

## Declaration of competing interest

The authors declare that they have no known competing financial interests or personal relationships that could have appeared to influence the work reported in this paper.
