# Supplementary material for: Demaghi, a polyherbal formulation, mitigates aluminum chloride-induced neurological impairment in mice: Insights from phytochemical analysis and behavioral assessment
Source: Heliyon. 2023 Oct 27;9(11):e21234. doi: 10.1016/j.heliyon.2023.e21234 (PMC10643107; doi:10.1016/j.heliyon.2023.e21234)
Supplement: Multimedia component 1 [file mmc1.docx]

**Demaghi, a polyherbal formulation, mitigates aluminum chloride-induced neurological impairment in mice: insights from phytochemical analysis and behavioral assessment**

^#^Hassan Ali^a^, ^#^Hafiz Usman^a^, Waseem Ashraf^a*^, Faleh Alqahtani^b^, Sana Javaid^a,c^, Farhan Siddique^d^, Muhammad Fawad Rasool^e^, Imran Imran^a^, Tanveer Ahmad^f^, Anas M. Abdel Rahman^g^, Reem H. AlMalki^h^

**Composition of Demaghi:**

| **Serial #** | **Ingredients of Demaghi** | **Scientific Name** | **Family** | **Parts or things used in formulation of Demaghi** |
| --- | --- | --- | --- | --- |
| 1 | Coral Compound | *Corallium species*  *Corallium rubrum* | *Corallidae* | Minerals |
| 2 | Borage | *Borago officinalis* | *Boraginaceae* | Seeds & whole fresh plant. |
| 3 | Clary Sage | *Salvia sclarea* | *Lamiaceae* | Herbs, seeds extract & essential oil. |
| 4 | Leopards Bane | *Doronicum hookeri,*  *Doronicum orientale* | *Asteraceae* | Flowers. |
| 5 | White Behen | *Centaurea behen* | *Asteraceae* | Roots. |
| 6 | Indian Catmint | *Anisomeles indica* | *Lamiaceae* | Leaves & Flowers. |
| 7 | Coriander | *Coriandrum sativum* | *Apiaceae* | Seeds & whole fresh plant. |
| 8 | Rose | *Rosa indica*  *Rosa damascena* | *Rosaceae* | Flowers, essential oils |
| 9 | Cinnamon | *Cinnamomum verum* | *Lauraceae* | Barks. |
| 10 | White Sandalwood | *Santalum album* | *Santalaceae* | Oily Extracts (resins) & whole fresh plant. |
| 11 | Lichen | *Parmotrema perlatum* | *Parmeliaceae* | Extracts / Minerals |
| 12 | Emblic | *Phyllanthus emblica* | *Phyllanthaceae* | Dried fruit |
| 13 | Purslane | *Portulaca oleracea* | *Portulacaceae* | Whole plant |
| 14 | Borage flowers | *Borago officinalis* | *Boraginaceae* | Flower, Leaves & whole fresh plant. |
| 15 | Bamboo Manna | *Bambusa species*  *Bambusa arundinacea* | *Poaceae* | Siliceous secretions |
| 16 | Barberry | *Berberis vulgaris* | *Berberidaceae* | Dried fruits & berries. |
| 17 | Olibanum Gum | *Boswellia Serrata* | *Burseraceae* | Oily extracts (Resins). |
| 18 | Cooling Seeds | *Ocimum basilicum*  *Ocimum tenuiflorum* | *Lamiaceae* | Seeds & whole fresh plant. |
| 19 | Virgenia peppergrass | *Lepidium virginicum* | *Brassicaceae* | Leaves, flowers & seeds extract. |
| 20 | Greater Cardamom Seed | *Amomum subulatum* | *Zingiberaceae* | Seeds |
| 21 | Screw Pine | *Pandanus odorifer Pandanus odoratissimus* | *Pandanaceae* | Extract from flowers. |
